# Loss of Uncoupling Protein 1 Expression in the Subcutaneous Adipose Tissue Predicts Childhood Obesity

**DOI:** 10.3390/ijms242316706

**Published:** 2023-11-24

**Authors:** Katalin Gyurina, Mariia Yarmak, László Sasi-Szabó, Sarolta Molnár, Gábor Méhes, Tamás Röszer

**Affiliations:** 1 Institute and University Clinics of Pediatrics, Faculty of Medicine, University of Debrecen, 4032 Debrecen, Hungarysasi.szabo.laszlo@med.unideb.hu (L.S.-S.); 2 Department of Pathology, Faculty of Medicine, University of Debrecen, 4032 Debrecen, Hungary; molnar.sarolta@med.unideb.hu (S.M.);; 3 Institute of Neurobiology, Ulm University, 89081 Ulm, Germany

**Keywords:** brown adipose tissue, thermogenesis, adiposity, childhood obesity, metabolism

## Abstract

Stimulation of thermogenesis by inducing uncoupling protein 1 (UCP1) expression in adipocytes is thought to promote weight loss by increasing energy expenditure, and it is postulated that the human newborn has thermogenic subcutaneous fat depots. However, it remains unclear whether a relevant number of UCP1-expressing (UCP1^+^) adipocytes exist in the early postnatal life. Here we studied the distribution of UCP1 and the expression of thermogenic genes in the subcutaneous adipose tissues of the human fetus, infant and child. We show that the deep layer of human fetal and neonatal subcutaneous fat, particularly the abdominal wall, is rich in UCP1^+^ adipocytes. These adipocytes develop in the late third trimester and persist throughout childhood, expressing a panel of genes linked to mitochondrial biogenesis and thermogenesis. During the early childhood adiposity rebound—a critical phase that determines obesity risk later in life—the absence of adipose tissue UCP1 expression in children with normal body mass index (BMI) correlates with an obesity-associated gene expression signature. Finally, UCP1 expression is negatively correlated with BMI *z*-score and adipocyte size in infants and children. Overall, our results show that the absence of UCP1 expression in adipose tissue is an early indicator of adipose tissue expansion in children.

## 1. Introduction

Childhood obesity is a significant public health concern that increases the likelihood of lifelong overweight or obesity and multiplies the risk of hypertension, cardiovascular disease, metabolic disorders, and diabetes in later life. Approximately 20% of children and adolescents had overweight or obesity in 2022 [1] and ~57% of children will become obese adults in the next decade [2]. The development of obesity is largely dependent on diet and lifestyle, and accelerated weight gain in the first year of life increases the likelihood of early obesity rebound in childhood, leading to permanent obesity by adolescence [3,4]. Adipose tissue expansion is progressive and can be reversed by early interventions. However, childhood obesity is commonly diagnosed when clinical signs—such as an increased body mass index (BMI)—are evident, and metabolic complications such as insulin resistance and diabetes can already be apparent [5,6]. At this stage, weight loss is challenging, in part because of the metabolic properties of the storage fat: once established, subcutaneous fat is stable and difficult to lose in children [7]. From a clinical perspective, it is thus crucial to identify diagnostic markers that indicate early-stage adipose tissue expansion, and are independent from BMI [8].

The discovery of adaptive thermogenesis in small rodents has led to the concept that inducing thermogenesis in human storage fat depots will enhance fat loss by increasing energy expenditure [9,10]. The process of adaptive thermogenesis involves the development of thermogenic adipocytes expressing uncoupling protein 1 (UCP1), often referred to as beige adipocytes, from precursors of storage fat adipocytes [11]. Termed “adipose tissue browning”, this process has attracted much attention because of its therapeutic potential [10,12,13]. Animal studies have shown that loss of UCP1-expressing subcutaneous adipocytes in early postnatal life predisposes to adult obesity [14,15,16]. It is postulated that the human newborn has interscapular thermogenic fat depots, similar to rodents [9]. Understanding the mechanisms that allow expansion of this thermogenic fat depot would facilitate obesity therapy [9]. However, this putative thermogenic fat depot was described before the discovery of UCP1 and the availability of immunohistochemistry [17]. Therefore, it remains unclear whether UCP1-expressing adipocytes constitute a relevant portion of the subcutaneous adipose tissues in the early postnatal life [18].

In the present study, we investigated the expression of UCP1 in human subcutaneous adipose tissue during intrauterine development and postnatal growth. Tissue specimens were collected from cases of perinatal fatalities and during elective surgery interventions. Samples were processed for histology and immunostaining for UCP1. Expression levels of UCP1 and gene products associated with thermogenesis and mitochondrial biogenesis were measured with quantitative PCR (qPCR) and next generation RNA sequencing. The most direct approaches to resolve childhood obesity are prevention and reversal of overweight at its early stage, but we lack diagnostic markers that can identify adipose tissue expansion before the onset of clinical signs of obesity. We intended to define whether there is value in using UCP1 protein or UCP1 mRNA expression in adipose tissue biopsies to identify early-stage adipose tissue expansion.

## 2. Results

### 2.1. Lack of a Thermogenic Interscapular Fat Depot in Human

**Figure 1:**
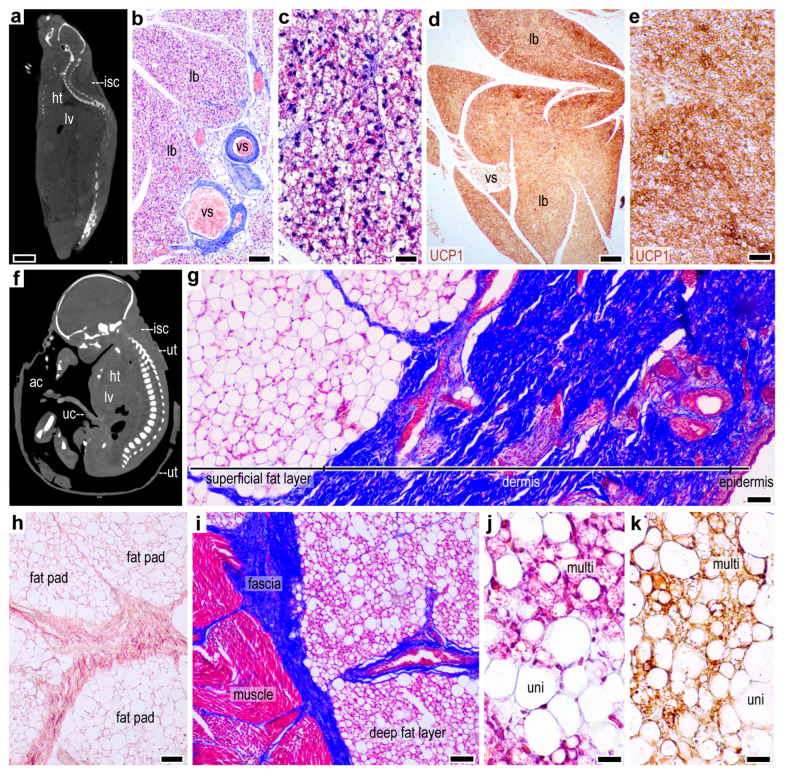
Comparison of mouse and human interscapular fat depots. (**a**) Sagittal computed tomography (CT) scan image of an adult C57/BL6 male mouse, showing the interscapular fat depot (isc); ht: heart, lv: liver, scale bar 0.7 cm. (**b**) Masson’s trichrome stained section of the mouse interscapular fat depot, showing densely arranged multilocular adipocytes in large lobes (lb), giving a gland-like appearance to the fat depot. vs: blood vessels, scale bar 100 μm. (**c**) Multilocular adipocytes in the mouse interscapular fat, Masson’s trichrome staining, scale bar 50 μm. (**d**) UCP1 immunostaining of the mouse interscapular fat; lb: lobes, vs: blood vessels, scale bar 100 μm. (**e**) UCP1-positive multilocular adipocytes in the mouse interscapular fat, scale bar 50 μm. (**f**) Sagittal CT scan image of a human fetus in utero at gestational week 36–38; isc: interscapular fat, ut: uterus wall, ht: heart, lv: liver, ac: amniotic cavity, uc: umbilical cord, head to toe length 47 cm. (**g**) Masson’s trichrome staining of the interscapular fat at gestational week 36, scale bar 100 μm. (**h**) Orcein staining of elastic fibers in the interscapular fat at gestational week 36, scale bar 100 μm. (**i**) Masson’s trichrome staining pf the perimuscular fat surrounding the trapezius muscle at gestational week 36, scale bar 50 μm. (**j**) Multilocular (multi) and unilocular (uni) adipocytes in the perimuscular fat, scale bar 30 μm. (**k**) UCP1 immunostaining of the perimuscular fat, scale bar 20 μm.

Since the first identification of thermogenic adipose tissue, it has been postulated that the interscapular region in the human infant is rich in thermogenic adipocytes [17]. To explore this idea, we searched for thermogenic adipocytes in this fat depot in comparison with the interscapular fat from mice, used as a positive control. In the mouse, the interscapular fat pad was highly vascularized with abundant multilocular adipocytes (Figure 1a–c) staining strongly for UCP1 (Figure 1d,e). Contrastingly, the interscapular fat pad in the human infant consisted solely of unilocular adipocytes lacking UCP1 immunopositivity (Figure S1).

We next examined the same region in the human fetus. The interscapular fat pad was detectable only in the third trimester (Figure S2) and became well established by the 36th week of gestation (Figure 1f,g). At this stage of development, the interscapular skin was composed of a thick collagenous dermis and a large subcutaneous fat layer (Figure 1g). The fat layer was composed of unilocular adipocytes, intersected by collagen and elastic fibers (Figure 1g,h). Notably, densely arranged multilocular adipocytes were detectable beneath this fat layer, around the fascia of the trapezius muscle, and were immunopositive for UCP1 (Figure 1j,k).

Overall, these findings show that, unlike in mice, the interscapular fat depot in the human fetus and infant is mostly a storage fat depot. However, thermogenic adipocytes are prevalent beneath this storage fat layer and form an epimuscular fat sheath. This layer is, however, located deeply and is not accessible by routine sampling in pediatric practice as no surgery is typically performed at this anatomical site, and hence it has no diagnostic value.

### 2.2. Subcutaneous Thermogenic Fat in the Abdominal Region

**Figure 2:**
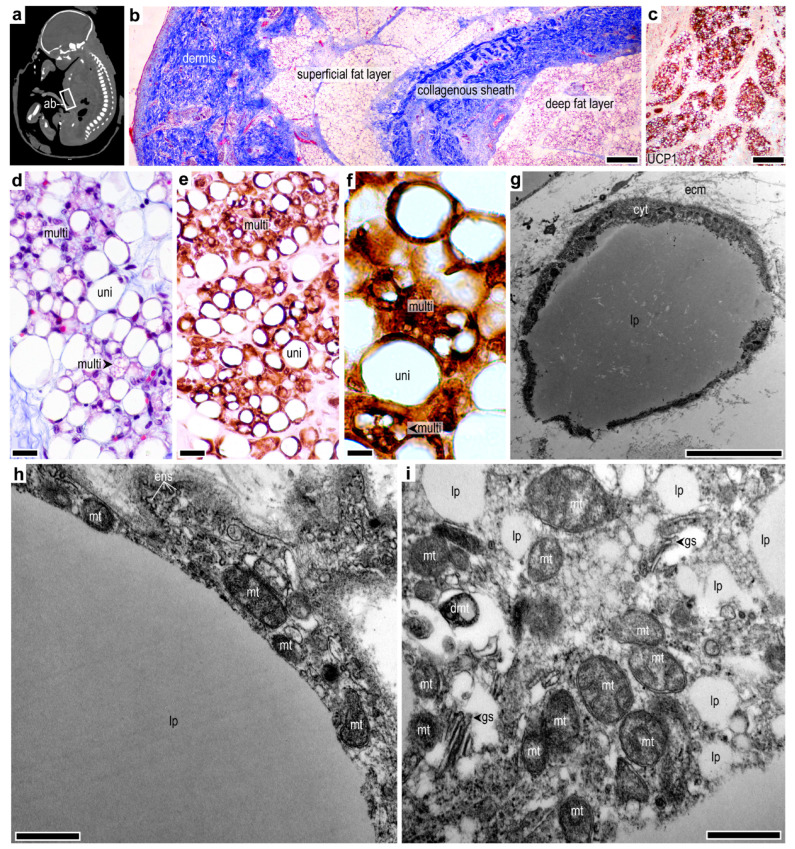
Thermogenic adipocytes in the human abdominal subcutaneous fat depot. (**a**) Sagittal computed tomography scan image showing the abdominal wall (ab) fat depot in a human fetus at gestational week 36–38, head to toe length 47 cm. (**b**) Masson’s trichrome staining of the abdominal subcutaneous fat depot at gestational week 36, scale bar 200 μm. (**c**) UCP1 immunostaining of the deep fat layer at gestational week 36. Scale bar 200 μm. (**d**) Clusters of multilocular adipocytes (multi) among unilocular adipocytes (uni) at gestational week 36, scale bar 50 μm. (**e**) UCP1 immunostaining of the region shown in panel (**d**), scale bar 30 μm. (**f**) UCP1 immunostaining of adjacent unilocular and multilocular adipocytes, lp: lipid droplet, scale bar 5 μm. (**g**) Transmission electron micrograph of an adipocyte in the abdominal subcutaneous fat depot in a 0.58-year-old infant. lp: lipid droplet, cyt: cytoplasm, ecm: extracellular matrix, scale bar 1 μm. (**h**) Transmission electron micrograph of a unilocular adipocyte, showing the central lipid droplet (lp) and portion of the cytoplasm. The cytoplasm is rich in mitochondria (mt), and the cell membrane generates endosomes (ens), scale bar 1 μm. (**i**) Cytoplasm of a multilocular adipocyte. mt: mitochondria, dmt: damaged mitochondria, gs: Golgi system, lp: lipid droplets. Scale bar 1 μm.

We next asked whether thermogenic adipocytes were present in other subcutaneous locations in the human fetus. We found that the abdominal skin contained multilocular, strongly UCP1-immunopositive adipocytes (Figure 2a–c). These adipocytes formed a fat layer that was separated by a collagenous connective tissue sheath from a superficial layer of subcutaneous storage fat (Figure 2b). The majority of the UCP1^+^ adipocytes had a multilocular morphology (Figure 2d–f). Moreover, UCP1^+^ adipocytes were prevalent in the deep layer of the abdominal subcutaneous fat depot after birth and persisted in childhood (Figure S3) and were rich in mitochondria, as determined by TEM (Figure 2g–i). Several lipid droplets were seen, as well as signs of endosome trafficking and active protein synthesis (Figure 2i). UCP1^+^ adipocytes were also detectable in the subcutaneous fat layers of the thigh but were absent from the fibrous fat pad of the foot sole (Figure S4).

### 2.3. Adipose Tissue UCP1 Is Associated with Thermogenic Gene Expression

**Figure 3:**
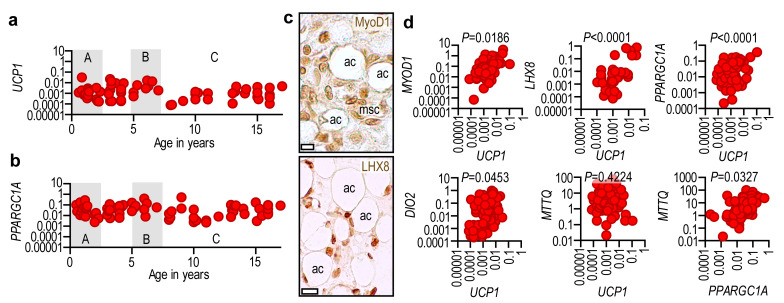
Association of UCP1 mRNA expression with thermogenic gene expression. (**a**) Expression level of UCP1 mRNA normalized to the expression level of ACTNB and GAPDH, in subcutaneous abdominal adipose tissue samples from patients aged 0.58–17 years (males: 72, females: 26). Each data point represents one patient. A: infancy, B: adiposity rebound in early childhood, C: pre-puberty and puberty. (**b**) Expression level of *PPARGC1A* mRNA normalized to the expression level of *ACTNB* and *GAPDH* in the same patients. (**c**) Immunohistochemistry of Myod1 and LHX8 in the abdominal subcutaneous fat at gestational week 36–38. ac: adipocyte, msc: mesenchymal cells, scale bar 5 μm. (**d**) Correlation of adipose tissue *UCP1* mRNA level with the mRNA level of genes necessary for thermogenic fat development, thermogenesis, and mitochondrial biogenesis. Same patients as in panels (**a**,**b**). Pearson’s 2-tailed correlation analysis with 95% confidence interval.

Expression analysis revealed that *UCP1* mRNA—which has been shown to reflect the UCP1 protein level [14,16]—peaked after birth and declined during the first two years of life (Figure 3a). A second peak occurred at the time of adiposity rebound (Figure 3a), after which *UCP1* expression declined but remained steady to young adulthood (Figure 3a). The expression pattern of *UCP1* was mirrored by the expression of *PPARGC1A* (Figure 3b), encoding PPAR gamma coactivator 1 alpha, the key regulator of mitochondrial biogenesis.

We next questioned whether *UCP1* mRNA expression correlated with other genes involved in thermogenesis and mitochondrial biogenesis. Using subcutaneous fat samples from the abdominal region of patients aged 0.5–17.3 years, we measured the mRNA levels of *UCP1*, *PPARGC1A, DIO2* (encoding iodothyronine deiodinase 2), *LHX8* (encoding LIM homeobox 8), *MYOD1* (encoding myogenic differentiation 1, MyoD1), and *MTTQ* (also known as *TrnQ*, which encodes the tRNA of glutamine in the mitochondrial genome). DIO2 has an important role in adipocyte browning, and LHX8 and MyoD1 are associated with preadipocytes that have thermogenic competence [13,19].

We used the level of *MTTQ* to assess the number of mitochondria in adipocytes. Immunohistochemistry analysis revealed that the abdominal wall fat layer was rich in MyoD1^+^ and LHX8^+^ adipocytes and mesenchymal cells (Figure 3c). We found a positive association between adipose tissue *UCP1* expression and *LHX8*, *MYOD1*, *PPARGC1A*, and *DIO2* mRNA levels (Figure 3d). While the correlation between *UCP1* and *MTTQ* levels was weak, *PPARGC1A* expression showed a positive correlation with *MTTQ* levels (Figure 3d).

### 2.4. Lack of UCP1 Is Associated with the Expression of Obesity-Associated Genes

**Figure 4:**
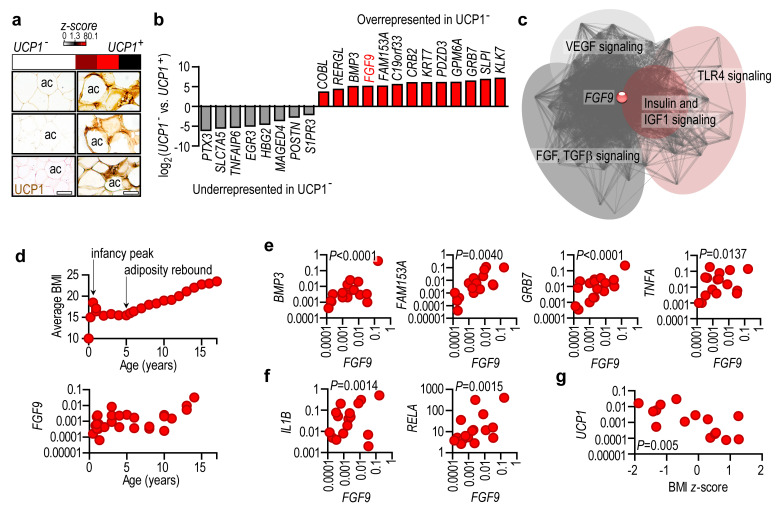
Lack of UCP1 is associated with a transcriptional signature of obesity. (**a**) Relative expression of *UCP1* mRNA and UCP1 immunostaining of six fat samples used for subsequent NGS analysis. ac: adipocyte, scale bar 30 μm. All samples were obtained from the lower abdominal region of male patients (mean age 5.3 years, mean BMI 19.3). (**b**) Differentially expressed genes in UCP1^+^ and UCP1^−^ samples shown in panel (**a**). (**c**) STRING protein–protein interaction map of FGF9-associated transcripts. VEGF: vascular endothelial growth factor, TLR: Toll-like receptor, FGF: fibroblast growth factor, IGF1: insulin-like growth factor 1. (**d**) *Top*: BMI trajectory of the study group. *Bottom*: *FGF9* mRNA level in subcutaneous abdominal adipose tissue samples from patients aged 0.58–17 years. (**e**) Correlation of *FGF9*, *FAM153A*, *BMP3*, and *TNFA* mRNA expression, (**f**) *FGF9*, *IL1B*, and *RELA* mRNA expression in subcutaneous abdominal adipose tissue samples from patients aged 0.58–17 years. (**g**) Correlation of BMI z-score and *UCP1* mRNA level in adipose tissue samples from patients aged 0.3–2 years (infancy peak of adiposity) and 4–8 years (adiposity rebound). Pearson’s 2-tailed correlation analysis with 95% confidence interval.

Adiposity rebound occurs physiologically at ~5.5 years of age and is a critical period that determines obesity risk later in life [20]. We selected three patients with undetectable *UCP1* mRNA levels in their fat and processed the same samples for histology, which confirmed the absence of UCP1^+^ cells (Figure 4a). We used these samples for next generation sequencing (NGS) and compared their transcriptional profiles with those of age- and sex-matched patients with detectable *UCP1* mRNA and protein (Figure 4a). Samples were obtained from the lower abdominal region of male patients (mean age 5.3 years, mean BMI 19.3).

Despite showing a normal donor BMI, the UCP1^-^ samples displayed an over-representation of transcripts associated with obesity (Figure 4b), including *FGF9* (fibroblast growth factor 9), *GRB7* (growth factor receptor-bound protein 7), *COBL* (cordon-bleu WH2 repeat protein), *GPM6A* (glycoprotein M6A), *KLK7* (kallikrein 7), *BMP3* (bone morphogenetic protein), and the human adipocyte-associated transcript *FAM153A* (encoding family with sequence similarity 153 member A).

For instance, FGF9 has a central role in interconnecting gene networks of adipose tissue expansion (Figure 4c). Indeed, FGF9 has been reported to inhibit thermogenesis in mice [21], but this effect is controversial in human adipocytes [22]. We found that *FGF9* expression mirrored the BMI trajectory of infants and children (Figure 4d), and *FGF9* levels positively associated with *BMP3*, *FAM153A*, and *GRB7* expression (Figure 4e). BMP3 is associated with preadipocyte proliferation [23], FAM153A is a yet-to-be characterized protein of human adipocytes, and GRB7 is associated with human obesity [24]. Notably, the levels of *GRB7* positively associated with *RELA* (encoding NFκB) and proinflammatory genes *TNFA* (encoding tumor necrosis factor alpha) and *IL1B* (encoding interleukin 1 beta) (Figure 4e,f). NGS analysis also revealed that UCP1^-^ samples had an under-representation of gene products that have been shown to protect from obesity. These include *PTX3* (encoding pentraxin 3) [25], *SLC7A* (encoding an L-type amino acid transporter) [26], *TNFAIP6* (encoding tumor necrosis factor alpha-induced protein 6) [27], and *POSTIN* (encoding periostin) [28] (Figure 4b).

Overall, our results show that the lack of UCP1 at the period of adiposity rebound is associated with a transcriptional landscape of obesity and adipose tissue inflammation. Supporting this, adipose tissue *UCP1* expression was negatively associated with BMI *z*-score in patients aged 4–8 years (Figure 4g).

### 2.5. Adipose Tissue UCP1 Expression Level Reflects Obesity Status

**Figure 5:**
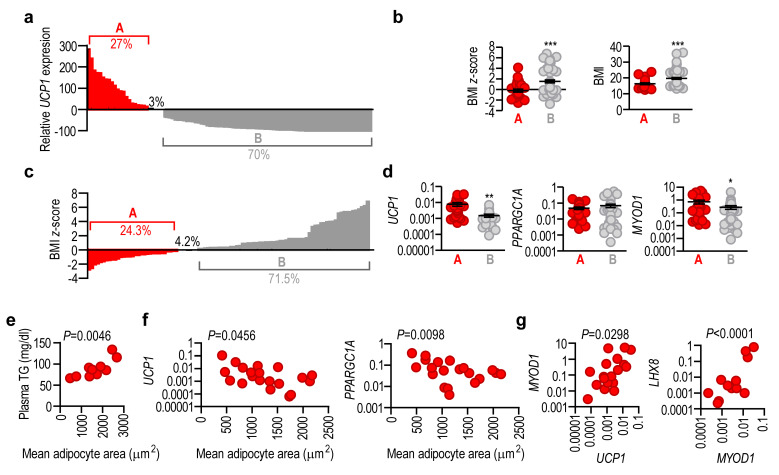
Association of *UCP1* mRNA expression with obesity status. (**a**) Relative adipose tissue *UCP1* mRNA levels (% of population mean of *UCP1*) in patients at 0.58–17 years of age. Each bar represents one patient: 27% of patients (group A) had an above average, while 70% of patients (group B) had a below average, *UCP1* mRNA level. (**b**) BMI *z*-score, BMI, and weight/height ratio of group A (above average *UCP1*) and group B (below average *UCP1*) patients. (**c**) BMI *z*-score values of the patient population shown in panel (**a**). A share of 24.3% of patients had a negative BMI *z*-score (group A), whereas 71.5% had a positive BMI *z*-score (group B). (**d**) Expression levels of *UCP1, PPARGC1A* and *MYOD1* in group A and group B patients. * *p* < 0.05, ** *p* < 0.001, *** *p* < 0.0001, Student’s 2-tailed unpaired *t*-test. (**e**) Correlation of mean adipocyte area and fasting plasma triglyceride (TG) levels in patients aged 0.2–6.5 years. (**f**) Correlation of mean adipocyte area with adipose tissue expression of *UCP1* and *PPARGC1A* mRNA in patients aged 0.2–6.5 years. (**g**) Correlation of *UCP1* with *MYOD1* and *LHX8* mRNA levels in the same samples show in panel (**f**). Pearson’s 2-tailed correlation analysis with 95% confidence interval.

We found that 27% of the study group had higher *UCP1* levels than the population average and 70% had lower levels (Figure 5a). Patients with below-average *UCP1* levels also had an increased BMI and BMI *z*-score (Figure 5b). Overall, 24.3% of the study group had a negative BMI *z*-score and 71.5% had a positive BMI *z*-score, mirroring the *UCP1* levels (Figure 5c). BMI *z*-scores were positively associated with mean adipocyte size (Figure S5), validating BMI *z*-scores as indicators of adiposity, and according to previous studies showing an increased adipocyte volume in pediatric obesity [29]. Adipose tissue expression of *UCP1* and *MYOD1* was lower in the patient population with a positive BMI *z*-score (Figure 5d), which was associated with a moderate but non-significant decrease in *PPARGC1A* expression. These results indicate that a negative deviation from the population average of *UCP1* level is reflected by overweight and obesity.

Up to 10% of infants and children are affected by undescended or retractile testes, testicular torsion, hydrocele, or inguinal and abdominal hernia [30,31]. In our validation cohort we questioned whether the abdominal wall adipose tissue removed during surgical resolution of these cases might be used for the diagnostic assessment of *UCP1* expression. We found that adipocyte size correlated positively with plasma triglyceride levels in infants and children aged 0.2–6.5 years (Figure 5e) and correlated negatively with adipose tissue *UCP1* and *PPARGC1A* levels (Figure 5f). In addition, adipose tissue *UCP1* levels were positively correlated with *MYOD1* and *LHX8* levels in infants and children aged 0.2–6.5 years (Figure 5g).

These findings collectively suggest that a reduced level of *UCP1* expression in the abdominal subcutaneous fat depot is an early indicator of adipose tissue expansion.

## 3. Discussion

**Figure 6:**
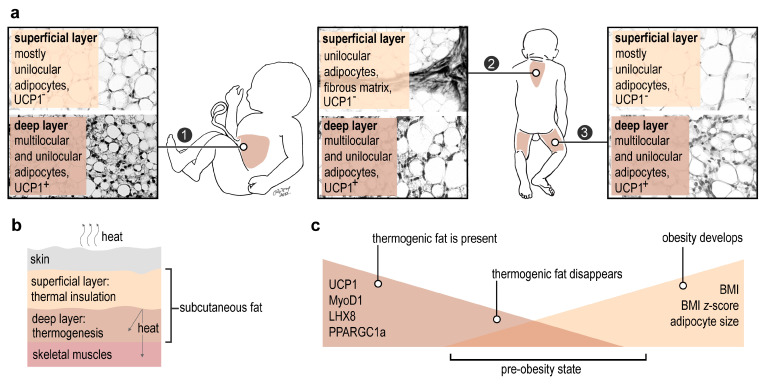
Impact of UCP1^+^ subcutaneous fat. (**a**) Distribution of thermogenic (UCP1^+^) subcutaneous fat in the human fetus and infant. 1: abdominal wall, 2: interscapular skin, 3: thigh. (**b**) Function of the UCP1^−^ and UCP1^+^ fat layers in human. (**c**) Diagnostic and prognostic potential of UCP1 in the subcutaneous fat.

We show here that the deep layer of human subcutaneous fat is rich in UCP1^+^ adipocytes starting from the last intrauterine trimester (Figure 6a). This UCP1^+^ fat layer forms a sheath around skeletal muscles and plausibly supports the core body temperature. The superficial subcutaneous fat layer is UCP1^-^, and most likely functions as a thermal insulator due to the low thermal conductivity of the stored triglycerides [32], to minimize heat loss through the skin (Figure 6b). Similarly widespread UCP1 expression has not been shown previously in the human subcutaneous adipose tissue, albeit sporadic data indicate UCP1 expression in the subcutaneous adipose tissue of human infants and children [14,18,33] and in adults [34,35]. It has been suggested by previous studies that the superficial and the deep layers of the subcutaneous fat have distinct functions and ontogeny [32,36]. Our findings corroborate a functional dichotomy of the dermal fat in the context of thermogenic competence and body weight control.

Brown adipose tissue is a thermogenic fat that was first described in the 16th century, long before the discovery of UCP1 and the development of molecular biology methodologies to measure UCP1 protein and mRNA [17]. In small mammals, UCP1-expressing adipocytes form a large interscapular fat depot, termed the interscapular brown adipose tissue [37,38,39]. In response to cold stress or to β-adrenergic stimulation, they also develop UCP1-expressing adipocytes in their subcutaneous fat, a process known as adaptive thermogenesis [40,41]. Birth marks a rapid increase in non-shivering thermogenesis [42,43,44] and an elevation in basal metabolic rate and oxygen consumption normalized to bodyweight [45,46]. There is evidence supporting the fact that almost 60% of the energy demand of non-shivering thermogenesis in human newborns is fueled by fat, and that the subcutaneous fat depots share morphological similarities with adipocytes from the interscapular fat of rodents [42,47,48,49,50].

There is a widespread assumption in the literature that the human newborn develops an equivalent of murine interscapular brown fat [9,17]; however, the supporting literature for this notion originated before the discovery of UCP1 [17]. Based on our findings, there is no rodent-equivalent interscapular brown fat pad in humans, although we detected a deep UCP1^+^ fat layer in the interscapular region surrounding the trapezius muscle in the human fetus. Instead, there is a large storage fat depot that may support the neck and back posture in an infant lying supine.

We corroborate the idea that thermogenic fat is not restricted to an interscapular depot, and is instead present throughout the subcutaneous fat in the human newborn, and is more extensive than was previously thought. Because the relative body surface of a human newborn is magnitudes greater than that of an adult, and because extrauterine existence begins with an adaptation to a hypothermic environment, it is plausible that the human newborn possesses the ability to generate heat in the subcutaneous fat, and this thermogenic competence is not limited to an interscapular fat layer as was assumed [17].

The thermogenic activation of adipose tissue is currently considered a feasible strategy to promote energy expenditure and force weight loss in obesity [9]. Most of the human thermogenic fat is, however, located around large vessels and vital internal organs that receive a rich supply of blood [13,40,51,52,53,54], and there is limited evidence of the presence of UCP1 expression in the adult subcutaneous adipose tissue [35,54,55]. The presence of a thermogenic fat sheath around vessels and vital organs minimizes heat loss of the circulating blood and supports the maintenance of core body temperature [56]. It remains an open question whether the expansion of these fat depots would contribute significantly to systemic energy expenditure and the loss of subcutaneous storage fat [17]. However, we show that having UCP1 expression within a storage fat depot in the sensitive period of early adipose tissue development is associated with a reduced adiposity. UCP1 expression hence may prevent an early adiposity rebound and the development of a BMI trajectory in childhood that may lead to obesity by adulthood.

The strengths of our study are the analysis of human adipose tissue specimens, direct approaches to detect and quantify UCP1, and describing the distribution of thermogenic fat from intrauterine development to puberty. In contrast, current methods to assess thermogenic human fat use in vivo imaging or in vitro techniques and focus on adult patients with obesity [53,54,57,58], without defining the precise anatomical distribution and ontogeny of UCP1-expressing fat. Because some fatty acids can uncouple mitochondrial respiration without the need for UCP1 [59], the thermogenesis that is detected by in vivo imaging may not indicate the existence of a true UCP1-expressing fat depot. Moreover, these imaging techniques have limitations in the pediatric practice. To date, only limited data are available on the distribution of UCP1-expressing subcutaneous adipocytes in the early postnatal life [18,34,57], making our findings relevant to understand the importance of UCP1 in the human storage fat depots.

Obesity reduces the thermogenic potential of the adipose tissue [35,52,57,58,60], polymorphic UCP1 increases the risk of liver disease in obese patients [61], and the premature loss of thermogenic potential is thought to favor storage fat development and potentially lead to childhood obesity [14,16,18]. We corroborate this possibility and provide evidence that the absence of UCP1 is concomitant with a compromised expression of mitobiogenesis and thermogenesis genes in the subcutaneous fat. Loss of adipose tissue *UCP1* before adiposity rebound is hence an early indicator of adipose tissue expansion. Although our study conclusions are limited by the fact that a specific patient population has been assessed, we have shown the negative correlation between *UCP1* mRNA and thermogenic gene expression in an unrelated patient population of the Leipzig Childhood Obesity Cohort [16]. Similarly, adult obesity has been shown to be associated with a reduced UCP1 expression in the subcutaneous adipose tissue [35].

From the perspective of the clinical utility of our findings, the absence of UCP1 is associated with the transcription of obesity-related genes, an increase in BMI *z*-score, greater adipocyte volume, and elevated plasma triglycerides. In addition to the over-representation of obesity-associated genes in UCP1^-^ samples, our NGS analysis identified a reduced expression of genes associated with obesity protection when UCP1 was absent. This makes it plausible that the absence of UCP1 increases the vulnerability to obesity. Moreover, the lack of UCP1 and associated gene expression changes precede the development of overweight (Figure 6c). In summary, absence of UCP1 not only hallmarks obesity, but importantly, UCP1 may disappear from the subcutaneous adipose tissue before the onset of overweight (Figure 6c). Some evidence suggests that efficiency of pharmacological interventions to increase adipose tissue thermogenesis depends on the volume of a functional thermogenic fat in humans [9]; therefore, knowing the UCP1 expression level in the adipose tissue may also aid decision making on personalized strategies of weight-loss management.

Overall, our results show that the absence of UCP1 expression in adipose tissue is an early indicator of adipose tissue expansion in children. Recognizing early-life adipose tissue expansion before the onset of clinical signs of overweight is critical to prevent further progression to obesity.

## 4. Materials and Methods

### 4.1. Human Samples

Fetal UCP1^+^ adipocytes were analyzed from adipose tissue specimens collected during autopsy of fetal death cases (Supplemental Table S1). Postmortem computed tomography (CT) scanning was performed on a museum specimen of an in utero male fetus [62]. Postnatal UCP1^+^ adipocytes were analyzed from adipose tissue specimens of 107 patients (male 79, female 28) of 0.2–17.3 years of age collected during elective surgery. All patients underwent elective surgery between June 2022 and September 2023 at the Institute and University Clinics of Pediatrics, University of Debrecen. Surgical indications were inguinal, umbilical, or abdominal hernia (both sexes), or an indication for orchidopexy in male patients. As a result of sex-dependent differences in the frequency of surgical interventions in early childhood, females were less represented than males in our study population. Selection and exclusion criteria were as described [14]. In brief, inclusion criteria included the appropriate surgical intervention that yielded adipose tissue as a surgical waste, and a written informed consent from the parent/guardian. Exclusion criteria were diabetes or immune conditions, genetic diseases, acute infection, recent history of COVID-19 disease, PCR-positivity for SARS-CoV-2, bleeding and hematological disorders, oncological disease, cachexia, maternal diabetes, or autoimmune diseases, and maternal PCR-positivity for SARS-CoV-2. All patients younger than 0.5 years of age had received human milk feeding at the time of surgery in the form of exclusive or partial breastfeeding. Fasting plasma triglycerides were determined in venous blood by a commercial kit (CliniChem Ltd., Budapest, Hungary), according to the manufacturer’s protocol. BMI and BMI *z*-score were determined according to guidelines from the World Health Organization, as previously described [3]. Mean BMI was 18.31 ± 0.59 for males and 17.23 ± 0.84 for females, and mean BMI *z*-score was 0.95 ± 0.28 for males and −0.10 ± 0.58 for females; distribution of individual BMI and BMI *z*-score values are indicated in the respective figures.

### 4.2. Animals

Adipose tissue specimens were collected from adult male C57/BL6 mice, as previously described [16]. A postmortem CT image was obtained by a scan performed on a small-animal multimodality imaging system (NanoSPECT-CT^®^, Mediso Ltd., Budapest, Hungary). Images were reconstructed using RadiAnt DICOM Viewer (Medixant, Poznan, Poland).

### 4.3. Histology, Immunohistochemistry, and Transmission Electron Microscopy

Tissues were fixed in 4% paraformaldehyde dissolved in PBS, embedded in paraffin, and cut with a microtome to obtain 7 μm thick sections. The general structure of the specimens was evaluated with hematoxylin and eosin and Masson’s trichrome staining. Adipocyte area was measured with ImageJ, as previously described [16]. Some sections were stained with orcein to visualize elastic fibers. For immunohistochemistry analysis, we used rabbit polyclonal antibodies against human UCP1 (1:250, PA1-24894, ThermoFisher Scientific, Rockford, IL, USA), LIM homeobox 8 (LHX8, 1:500, PA5-102563, Invitrogen, ThermoFisher Scientific) and myogenic differentiation 1 (MyoD1, 1:1000, SAB4300397, Merck Millipore, Darmstadt, Germany). Antibody binding was revealed with an HRP-conjugated secondary antibody and staining with Impact DAB (Vector Labs, Burlingame, CA, USA). Specificity of UCP1 immunostaining has been previously validated [16]. For transmission electron microscopy (TEM) analysis, samples were fixed in PFA-glutaraldehyde and processed, as previously described [14].

### 4.4. Gene Expression Analysis

Extraction of total RNA from adipose tissue was performed using TRIzol reagent (Merck Sigma-Aldrich, St. Louis, MO, USA), as previously described [16]. qPCR assays were carried out on a Quantabio platform (Beverly, MA, USA) and an Analytik Jena platform (Jena, Germany), using the mean threshold cycle (CT) value for *ACTINB* and *GAPDH* as a reference, expressing relative gene expression in logarithmic format. Primer sequences are shown in Supplemental Table S2. Next generation sequencing was performed and analyzed, as previously described [14], on a BGISEQ-500 platform by BGI Genomics Inc. (Cambridge, MA, USA). The difference between groups was determined by the log2 transformed fold change. Protein–protein interactome analysis was performed using STRING Functional Protein Associations Network, as previously described [14]. The dataset is available in NIH GEO under accession number GSE246973.

### 4.5. Statistical Analysis

Data are represented as mean and S.E.M. Data analysis and visualization was performed using GraphPad Prism 5.0 statistical software (San Diego, CA, USA). A one-matrix clustered image map (heat map) was generated with CIM Miner (https://discover.nci.nih.gov/cimminer/ (accessed on 20 November 2023)), using the Euclidean distance algorithm, average linkage cluster method, and equal width binning method. Study group size was determined with power analysis. Statistical differences were determined between groups with a normal distribution using the unpaired, 2-tailed Student’s *t*-test. Correlation analysis between two variables was performed using Gaussian *p*-value approximation with the 2-tailed Pearson test. The confidence interval was set at 95% (alpha = 0.05). The applied statistical tests, number of biological replicates, and *p*-values are defined in the respective figures and figure legends. In NGS analysis the sequencing data saturation analysis was used to measure whether the depth of sequencing data was sufficient for bioinformatics analysis. Based on their gene expression levels, we identified differentially expressed genes (DEGs) between sample groups, using the *DEseq2* algorithm to detect DEGs (*p* < 0.05) [63].

## 5. Conclusions

Our findings close a gap in our understanding of thermogenic fat development in the human fetus and newborn. First, we show that the UCP1^+^ subcutaneous fat develops in the late third trimester, and hence is not a response to the extrauterine exposure to the hypothermic environment.

UCP1 expression of the adipose tissue peaks at a sensitive early-life period that determines BMI trajectory in childhood. UCP1 expression hence appears in utero, and UCP1 remains persistent for a prolonged period of time in children. We also show that the human subcutaneous fat is rich in adipocytes with thermogenic competence, which are not limited to specific regions as was previously thought.

As a clinically relevant outcome of this study, we also show that lack of adipose tissue UCP1 expression in children is an early indicator of adipose tissue expansion and could be used to identify obesity risk before the onset of clinical signs of obesity.

It is anticipated that tissue-based diagnostics will promote personalized medicine in the future. UCP1 protein can be easily detected in fat samples removed during elective surgery and used as a diagnostic marker. Assessing UCP1 and thermogenic gene products (e.g., MyoD1, LHX8, DIO2, PPARGC1A) in tissue materials removed during elective surgery may be used to predict obesity risk and initiate interventions to mitigate further adipose tissue expansion at an early stage.

## Data Availability

Histology images are available in Figshare upon request; NGS datasets in NIH GEO (accession number: GSE246973).

## Supplementary Materials

The following supporting information can be downloaded at: https://www.mdpi.com/article/10.3390/ijms242316706/s1.
