# Supplementary material for: Loss of Uncoupling Protein 1 Expression in the Subcutaneous Adipose Tissue Predicts Childhood Obesity
Source: Int J Mol Sci. 2023 Nov 24;24(23):16706. doi: 10.3390/ijms242316706 (PMC10706300; doi:10.3390/ijms242316706)
Supplement: Supplementary file 1 [file ijms-24-16706-s001.zip › ijms-2737194-supplementary.pdf]

Supplemental Table S1. Characteristics of the perinatal fatalities analyzed in this study*

**Gestational age**	**Sex**	**Cause of death**
20 weeks	male	chorioamnionitis
20–23 weeks	male	nuchal cord, chorioamnionitis
23 weeks	female	stillborn, chorioamnionitis
30.3 weeks	male	infarctus placentae
34.6 weeks	male	chorioamnionitis
36–38 weeks	male	intrauterine asphyxia<br>interpositional cord insertion
36–37 weeks	female	placental abruption

* All patients were free from developmental malformations and indications of genetic disorders.

Supplemental Table S2. qPCR primer sequences used in this study

ACTB	fw	CACCATTGGCAATGAGCGGTTC
	rev	AGGTCTTTGCGGATGTCCACGT
GAPDH	fw	GTCTCCTCTGACTTCAACAGCG
	rev	ACCACCCTGTTGCTGTAGCCAA
UCP1	fw	AGTTCCTCACCGCAGGGAAAGA
	rev	GTAGCGAGGTTTGATTCCGTGG
MYOD1	fw	CTCCAACTGCTCCGACGGCAT
	rev	ACAGGCAGTCTAGGCTCGACAC
PPARGC1A	fw	CCAAAGGATGCGCTCTCGTTCA
	rev	CGGTGTCTGTAGTGGCTTGACT
DIO2	fw	TTGAGCCGCTCCAAGTCCACTC
	rev	CTGTACTGGAGACATGCACCAC
MT-TQ	fw	GATGTCAGAGGGGTGCCTTG
	rev	AACCCTCGTTCCACAGAAGC
LHX8	fw	GGACCAGCTTTACAGCAGATCAG
	rev	CGTCTGCTCAAGCCTGTCCTTT
FGF9	fw	CCAGGAAAGACCACAGCCGATT
	rev	CCATACAGCTCCCCCTTCTCAT
BMP3	fw	AAGGCAACACGGTTCGCAGCTT
	rev	GACTTGGTTAGCGATGTCAGATTG
FAM153A	fw	CAGACAGTCTCTGAGGAAGCCA
	rev	CTCACCGTTGTAACTGGACAGC
GRB7	fw	ACTCCACCAAGGGCACCTCTAA
	rev	GACACAGAAACCGAAGTCAGTGG

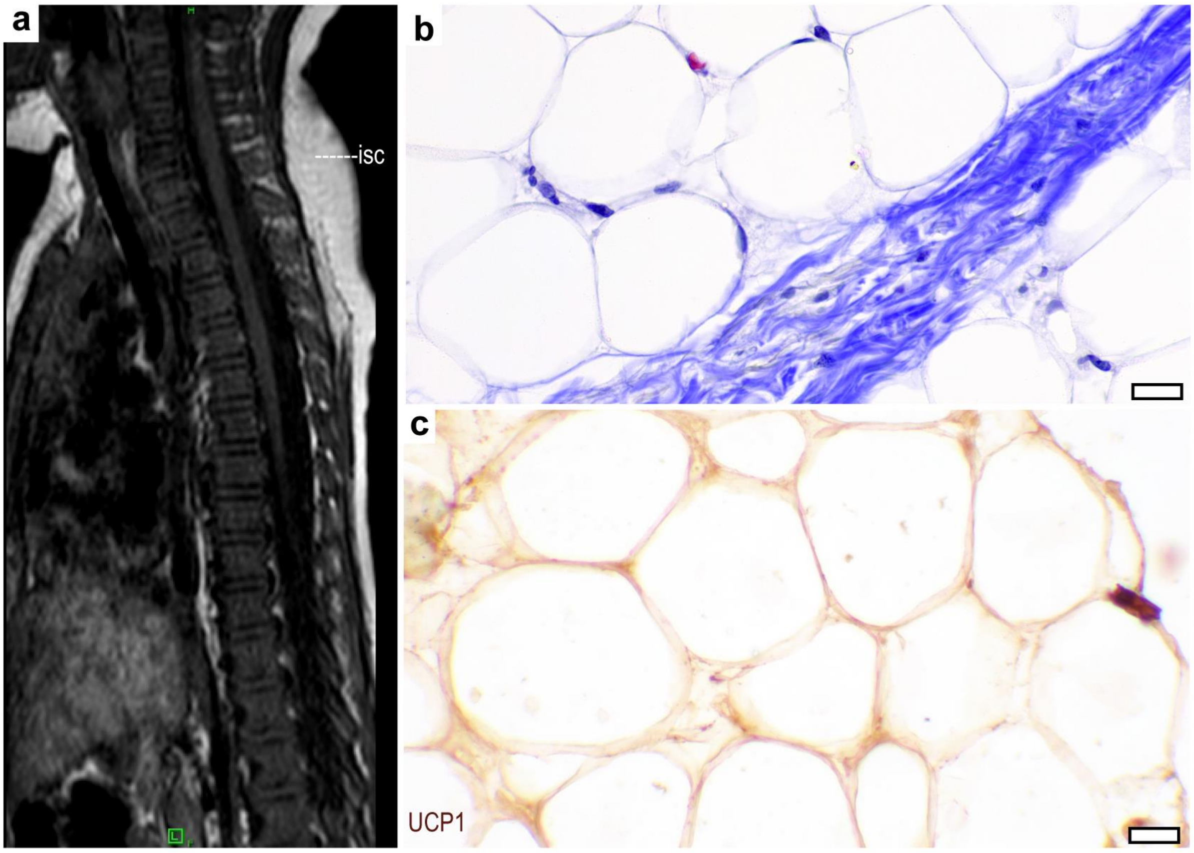

Figure S1. Interscapular fat depot in the human infant
(**a**) Sagittal MRI image of a 0.59-year-old infant, showing the position of the interscapular fat (isc). Courtesy of Dr. Veronika Gaál, University of Debrecen. (**b**) Masson's trichrome staining of the interscapular fat of a 1-year-old child. Scale bar 30 μm. (**c**) UCP1 immunostaining of the same sample. Scale bar 30 μm.

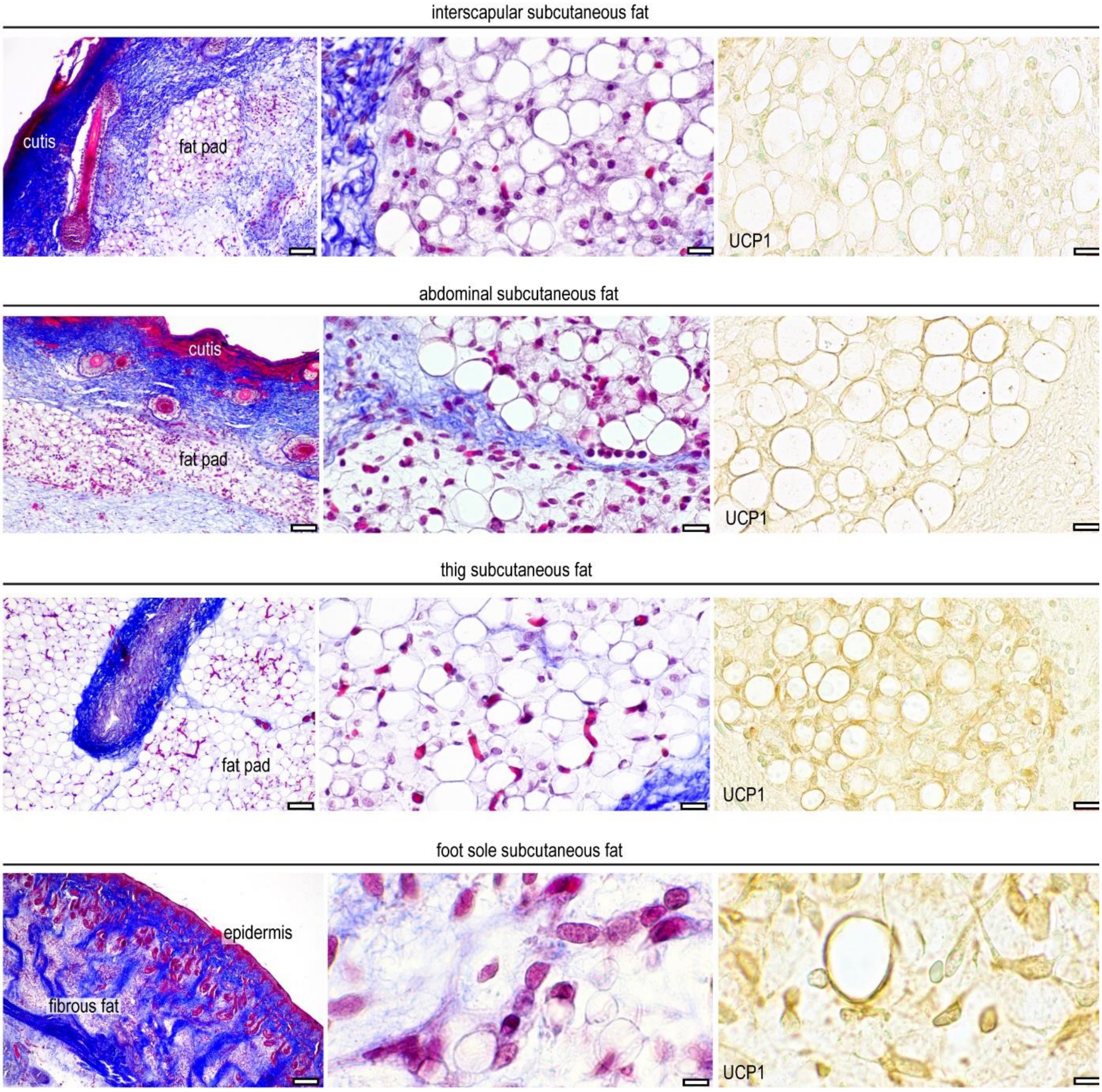

Figure S2. Fat depots in the second trimester

Representative Masson's trichrome and UCP1 immunostaining of various fat depots from the second trimester. Scale bar 50 μm.

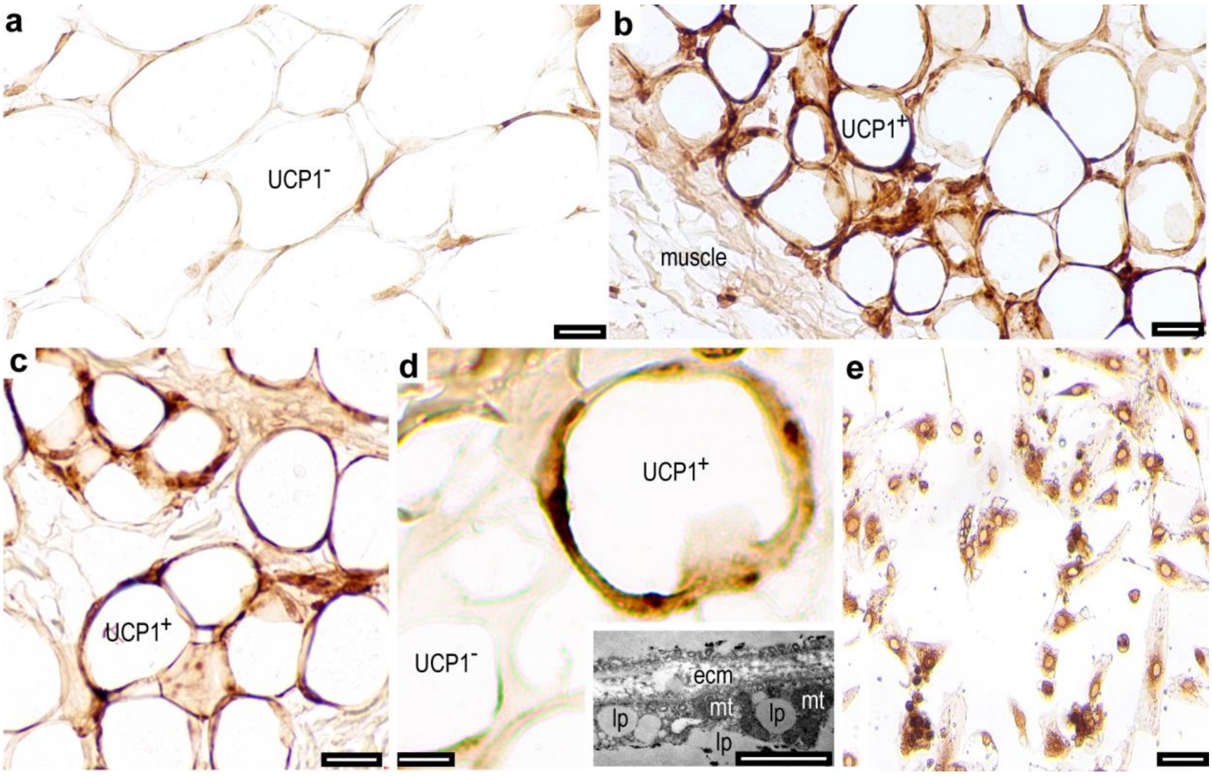

Figure S3. UCP1 immunostaining of the abdominal wall adipose tissue

(**a**) Superficial layer of the abdominal wall adipose tissue of a 6-year-old child. UCP1 immunostaining, scale bar 30 μm. (**b**) Deep layer of the abdominal wall adipose tissue of the same patient. UCP1 immunostaining, scale bar 30 μm. (**c**) Deep layer of the abdominal wall adipose tissue of a newborn. UCP1 immunostaining, scale bar 30 μm. (**d**) Unilocular UCP1^{+} and UCP1^{-} adipocytes from the abdominal wall adipose tissue of a newborn, scale bar 10 μm. *Insert:* Periphery of a unilocular adipocyte. The cytoplasm contains mitochondria (mt) and small lipid droplets (lp). ecm: extracellular matrix. Scale bar 0.5 μm. (**e**) UCP1 immunostaining of *in vitro* cultured adipocytes from the inguinal subcutaneous fat from a male patient (6.5 years old). Scale bar 50 μm. Cells were cultured as described (1, 2).

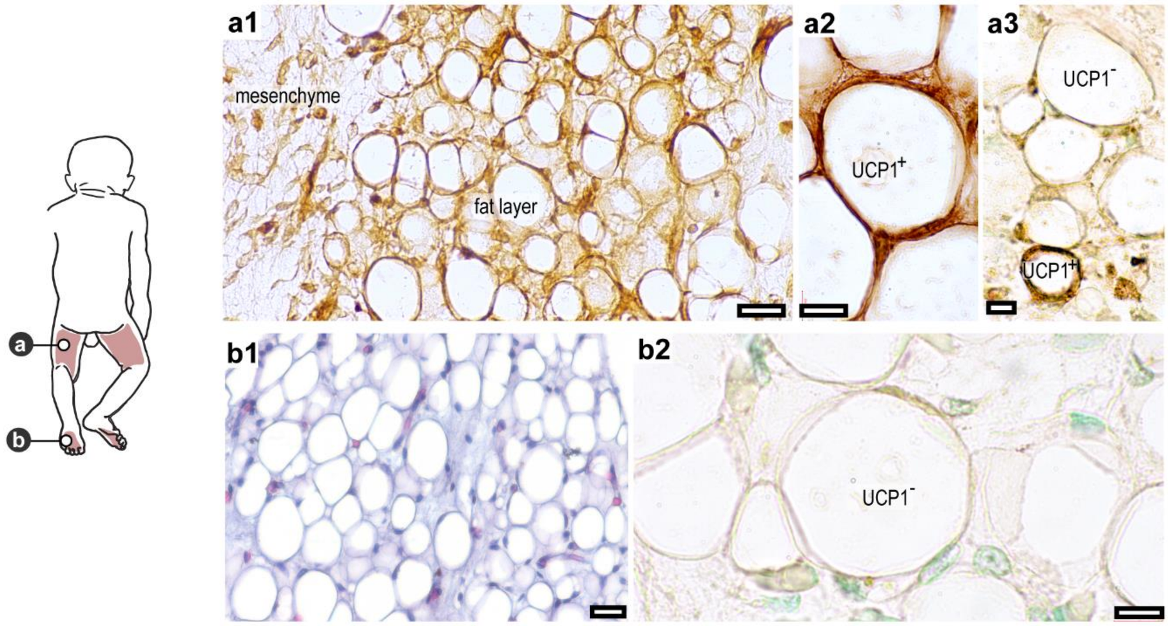

Figure S4. Fat depot of the thigh and the foot sole in the third trimester
(**a**) Thigh, (**b**) foot sole. **a1-a3**: UCP1 immunostaining, scale bar 100, 10, and 10 μm, respectively. **b1**: Masson's trichrome staining, scale bar 50 μm. **b2**: UCP1 immunostaining, scale bar 10 μm.

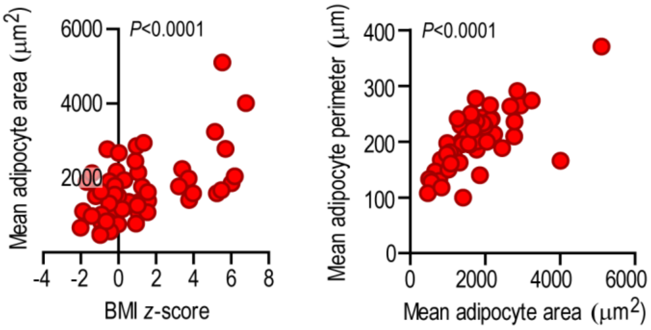

Figure S5. Correlation of mean adipocyte size and BMI *z*-score

Left: Correlation of mean adipocyte area measured in tissue sections and the corresponding BMI *z*-scores of the tissue donor patients. Each data point represents one patient. *Right:* Correlation of mean adipocyte area and perimeter. Imaging of the fat depots, such as CT or DEXA scanning cannot be employed in infants and children, hence we decided to use BMI *z*-score to estimate adiposity, according to WHO guidelines. Each data point represents one patient. Pearson's 2-tailed correlation analysis with 95% confidence interval.

References

1. Yu H, Dilbaz S, Coßmann J, Hoang AC, Diedrich V, Herwig A*, et al.* Breast milk alkylglycerols sustain beige adipocytes through adipose tissue macrophages. *The Journal of Clinical Investigation* 2019;**129:** 2485-2499.

2. Hoang AC, Sasi-Szabó L, Pál T, Szabó T, Diedrich V, Herwig A*, et al.* Mitochondrial RNA stimulates beige adipocyte development in young mice. *Nature Metabolism* 2022;**4:** 1684-1696.
